# Risk factors and antifungal resistance in *Candidozyma auris* bloodstream infections in Southern China

**DOI:** 10.1186/s12866-026-05523-5

**Published:** 2026-08-12

**Authors:** Siting Yi, Zetao Chen, Rong Wu, Fengying Wu, Jing Zhang, Pingjuan Liu

**Affiliations:** 1 Department of Laboratory Medicine, The First Affiliated Hospital, Sun Yat-sen University, Guangzhou, Guangdong 510080 China; 2 Department of Dermatology, Sun Yat-Sen Memorial Hospital, Sun Yat-Sen University, Guangzhou, Guangdong 510120 China; 3 Guangdong Provincial Key Laboratory of Malignant Tumor Epigenetics and Gene Regulation, Medical Research Center, Sun Yat-Sen Memorial Hospital, Sun Yat-Sen University, Guangzhou, Guangdong 510120 China; 4 Department of Dermatology, The Sixth Affiliated Hospital, Sun Yat-sen University, Guangzhou, Guangdong 510655 China; 5 School of Medical Technology, Guangdong Medical University, Dongguan, Guangdong 523808 China; 6 Dongguan Key Laboratory of Medical Bioactive Molecular Developmental and Translational Research, Guangdong Provincial Key Laboratory of Medical Immunology and Molecular Diagnostics, Guangdong Medical University, Dongguan, Guangdong 523808 China

**Keywords:** *Candidozyma auris*, Bloodstream infections, Risk factors, Antifungal resistance

## Abstract

**Background:**

*Candidozyma auris*, also known as *Candida auris*, is an emerging multidrug-resistant fungal pathogen that represents a major global health concern; however, its risk factors and antifungal resistance patterns in bloodstream infections (BSIs) in Southern China remain insufficiently characterized. Therefore, this study aimed to identify risk factors and antifungal resistance patterns associated with *C. auris* BSIs.

**Methods:**

A retrospective analysis was performed on 137 patients with candidemia at a large tertiary hospital in Southern China between October 2023 and September 2025. Least absolute shrinkage and selection operator regression followed by multivariate logistic regression was used to identify independent risk factors, and antifungal susceptibility testing was performed. Minimum inhibitory concentrations were determined and interpreted in accordance with the Clinical and Laboratory Standards Institute M27-A3 guideline.

**Results:**

*C. auris* was identified as the predominant pathogen, accounting for 39.4% (54/137) of candidemia cases, indicating an evident regional predominance and substantial burden. Independent risk factors included hypertension (OR = 6.283, 95% CI: 2.466–17.719), mechanical ventilation (OR = 4.329, 95% CI: 1.585–12.980), use of two or more antibiotics at infection onset (OR = 5.991, 95% CI: 2.349–16.686), and glucocorticoid therapy (OR = 3.100, 95% CI: 1.196–8.509). The 30-day mortality rate associated with *C. auris* infection was 18.5%. Lower C-reactive protein levels were independently associated with *C. auris* BSIs (OR = 0.987, 95% CI: 0.978–0.995), potentially reflecting a distinct host inflammatory response rather than representing a predisposing risk factor. Antifungal susceptibility testing revealed universal resistance to fluconazole (100%), while susceptibility to echinocandins was retained.

**Conclusions:**

This study identified *C. auris* as an emerging leading cause of candidemia in this region, characterized by different clinical risk factors and high antifungal resistance, highlighting the importance of early identification and targeted infection control strategies.

## Introduction

Since its first identification in 2009 from the ear canal discharge of a Japanese patient [1], *Candidozyma auris* (also known as *Candida auris*, or *C. auris*) has been recognized as an emerging and rapidly evolving global public health threat. It is characterized by efficient contact transmission and is associated with healthcare-related outbreaks in more than 50 countries across six continents as of 2025 [2, 3]. The recent isolation of this pathogen from Antarctica, as reported by van Rhijn et al., confirms its current presence on all seven continents [4]. A substantial and increasing proportion of mortality attributed to invasive fungal infections has been linked to *C. auris* worldwide [5]. In particular, bloodstream infections (BSIs) caused by *C. auris* are associated with considerable clinical burden, with reported attributable mortality rates ranging from 30% to 60% [6, 7]. Therefore, the effective control of *C. auris* BSIs remains a major clinical priority.

*C. auris* is readily transmitted through contact and is capable of persistent colonization in healthcare environments. Colonizing strains subsequently invade, leading to BSIs especially in high-risk populations, including patients in intensive care units, long-term care facilities, and immunocompromised individuals [8, 9]. Several clinical risk factors for *C. auris* infection have been reported, including exposure to broad-spectrum antimicrobial agents, central venous catheterization, mechanical ventilation, and glucocorticoid therapy [10, 11]. However, the first clinical isolate of *C. auris* in China was not reported until 2018 [12]. This delay may be closely related to the challenges associated with the routine identification of this fungus. Conventional biochemical identification systems frequently fail to identify *C. auris* or misidentify it as another fungal species because of their limited discriminatory power, whereas accurate identification requires advanced methods such as matrix-assisted laser desorption/ionization time-of-flight mass spectrometry (MALDI-TOF MS) or gene sequencing [13]. Nevertheless, some healthcare facilities in South China still lack access to these technologies, resulting in limited diagnostic capacity. Consequently, the relatively small number of cases reported in this region may be attributable to the limited availability of advanced diagnostic tools.

The high mortality associated with *C. auris* BSIs is largely attributed to its high level of antifungal resistance. A prevalence of fluconazole resistance of 91% has been reported across more than 4,000 cases in 33 countries [14], which substantially limits available therapeutic options [6, 15, 16] and contributes to nosocomial transmission. Nevertheless, evidence regarding the antifungal resistance patterns of *C. auris* in China remains limited. Thus, a retrospective analysis of candidemia cases identified at a tertiary hospital in Southern China was performed between October 2023 and September 2025 to address this gap and investigate the incidence, risk factors, and antifungal resistance profiles of *C. auris* BSIs, thereby providing clinically relevant evidence to support prevention and management strategies.

## Materials and methods

### Study design and patient selection

A retrospective analysis was performed on 137 adult patients diagnosed with candidemia at the First Affiliated Hospital of Sun Yat-sen University, a major tertiary care center in southern China, between October 2023 and September 2025. Only the first bloodstream isolate per patient was considered for analysis to reduce potential bias associated with recurrent or persistent infections.

**Fig. 1:**
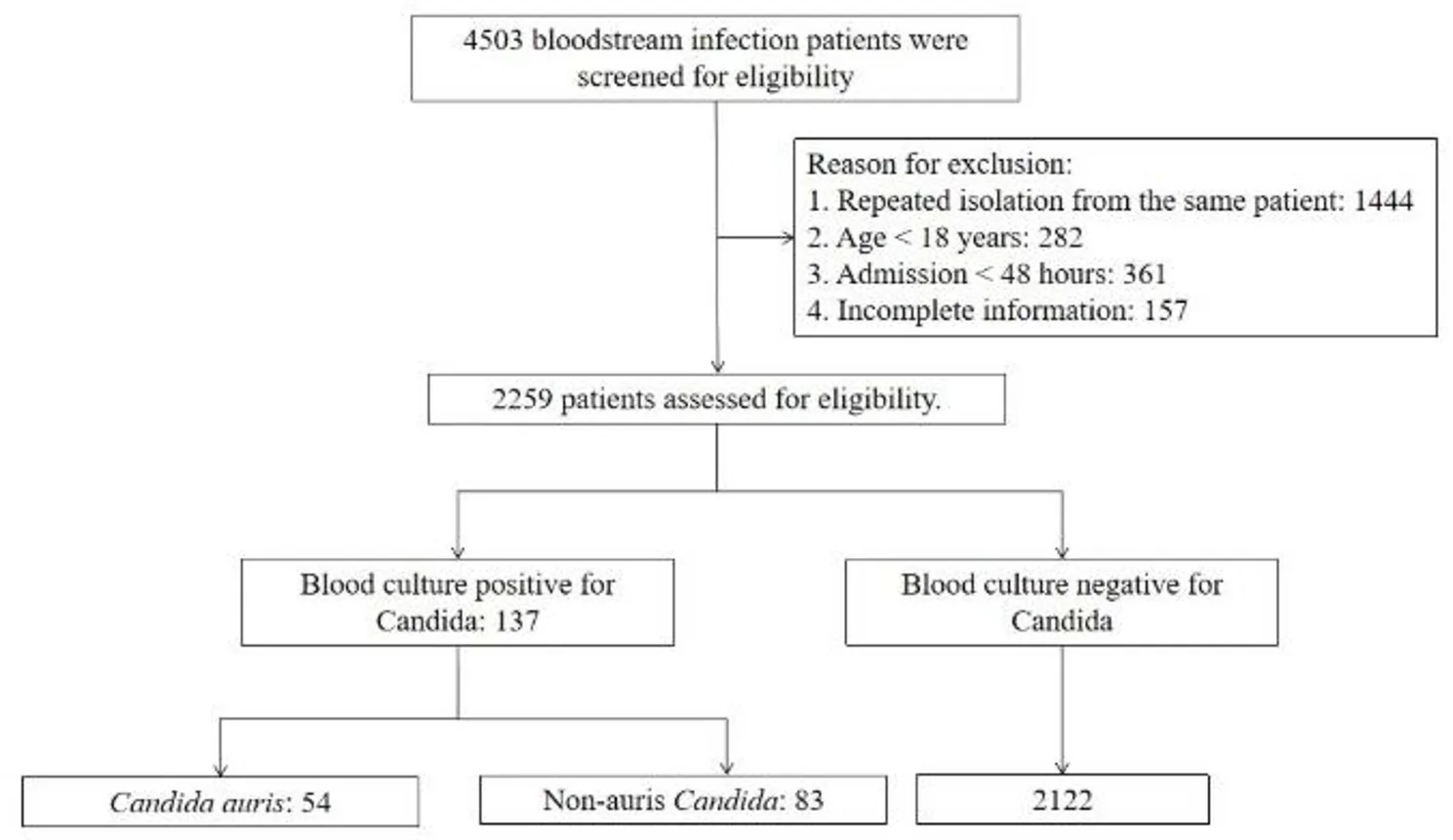
Flow diagram of patient enrollment

The inclusion criteria were the following: (1) age ≥ 18 years; (2) confirmed candidemia, defined by at least one blood culture positive for a single *Candida* species accompanied by clinical evidence of systemic inflammatory response, including body temperature ≥ 38 ℃ or < 36 ℃, chills, systolic blood pressure reduction > 40 mmHg from baseline or < 90 mmHg, heart rate > 90 beats/min, respiratory rate > 20 breaths/min, or altered mental status; (3) isolation exclusively of *Candida* species from blood culture; (4) availability of complete medical records. The exclusion criteria were the following: (1) hospital stay < 48 h; (2) identification of polymicrobial BSIs. Clinical and demographic characteristics, treatment-related variables, and clinical outcomes were systematically collected for all included patients. A schematic overview of the patient selection process is provided in Fig. 1.

### Microbiological methods

Blood culture samples were processed using the automated BacT/ALERT VIRTUO microbial detection system (bioMérieux, France) for continuous monitoring of microbial growth. Positive isolates were identified by MS (bioMérieux, France, V3.2). Antifungal susceptibility testing was performed using the broth microdilution method (Autobio, Zhengzhou, China). Minimum inhibitory concentration (MIC) values were determined and interpreted in accordance with the Clinical and Laboratory Standards Institute (CLSI) M27-A3 guidelines [17]. The MIC endpoint was defined as 50% growth inhibition for azoles and echinocandins, and 100% growth inhibition for amphotericin B (AMB). Specifically, MICs were read according to the manufacturer’s instructions: the MIC for AMB was defined as the lowest concentration at which the well remained blue or purple, indicating no color change compared to the positive control; the MIC for azoles, and echinocandins was defined as the lowest concentration producing a slight change in color intensity and a substantial reduction in precipitate (> 50%) relative to the positive growth control. All results were interpreted by experienced microbiologists in accordance with standard laboratory protocols.

Resistance was defined using the tentative CDC breakpoints as follows: a fluconazole MIC ≥ 32 µg/mL, an AMB MIC ≥ 2 µg/mL, and an echinocandins MIC ≥ 4 µg/mL. *Candida parapsilosis* (ATCC 22019) and *Pichia kudriavzevii* (formerly *Candida krusei*, ATCC 6258) were used as quality-control strains. The study protocol was reviewed and approved by the Institutional Ethics Committee of The First Affiliated Hospital of Sun Yat-sen University (No. [2025]503) and performed in accordance with the Declaration of Helsinki.

### Statistical analysis

The fungal isolates and their antifungal susceptibility data were analyzed using WHONET software (version 2025, World Health Organization). Statistical analysis was performed using R software (version 4.4.3). Continuous variables were assessed for distributional characteristics. Variables following a normal distribution were expressed as mean ± standard deviation and compared between groups using the independent samples *t*-tests. Non-normally distributed continuous variables were expressed as median (interquartile range [IQR]) and compared using the Mann–Whitney U test. Categorical variables were expressed as frequencies and percentages, and comparisons between groups were performed using the chi-square test. Least absolute shrinkage and selection operator (LASSO) regression was applied for preliminary variable selection. Given the sample size (*n* = 137), LASSO was used to reduce the risk of overfitting and select a parsimonious set of predictors from the 15 candidate variables. Variables identified at the one-standard-error criterion (λ.1se) were considered candidate predictors and subsequently included in a multivariate logistic regression model to determine independent risk factors. A value of *P* < 0.05 was considered statistically significant.

## Results

### Study population and baseline characteristics

A total of 137 adult patients with candidemia were enrolled in this study. The mean age was 61.01 ± 15.17 years, with a predominance of male patients (86, 62.8%). The most common comorbidities were chronic pulmonary disease (48.2%), hypertension (43.8%), and cardiovascular disease (40.1%), suggesting a substantial burden of underlying chronic conditions in this cohort. A high proportion of patients required mechanical ventilation (63.5%) or underwent surgical intervention (61.3%), reflecting the severity of the clinical status. Glucocorticoid therapy was administered in 56.2% of patients prior to the onset of candidemia, while 21.2% received echinocandins, and 15.3% triazole antifungal agents. Baseline patient characteristics are summarized in Table 1.

**Table 1:** Clinical characteristics of patients with *Candida* BSIs

Variable	Total	C. auris	Non-auris Candida	*P* value
	(*N* = 137)	(*N* = 54)	(*N* = 83)	
Male, *N* (%)	86 (62.8)	32 (59.3)	54 (65.1)	0.613
Age, Mean ± SD	61.01 ± 15.17	62.94 ± 14.75	59.76 ± 15.39	0.227
Comorbidities, *N* (%)				
Hypertension	60 (43.8)	35 (64.8)	25 (30.1)	< 0.001
Diabetes	50 (36.5)	23 (42.6)	27 (32.5)	0.311
Chronic pulmonary diseases	66 (48.2)	22 (40.7)	44 (53.0)	0.219
Cardiovascular disease	55 (40.1)	28 (51.9)	27 (32.4)	0.038
Cerebrovascular disease	30 (21.9)	14 (25.9)	16 (19.3)	0.479
Gastrointestinal disease	46 (33.6)	19 (35.2)	27 (32.5)	0.891
Renal dysfunction	42 (30.7)	17 (31.5)	25 (30.1)	1.000
Hepatic dysfunction	36 (26.3)	15 (27.8)	21 (25.3)	0.902
Solid organ tumor	32 (23.4)	9 (16.7)	23 (27.7)	0.198
Hypoalbuminemia	34 (24.8)	15 (27.8)	19 (22.9)	0.657
Invasive operation, *N* (%)				
Mechanical ventilation	87 (63.5)	46 (85.2)	41 (49.4)	< 0.001
Surgery	84 (61.3)	34 (63.0)	50 (60.2)	0.889
Previous drug exposure, *N* (%)				
Echinocandin	29 (21.2)	15 (27.8)	14 (16.9)	0.189
Triazoles	21 (15.3)	10 (18.5)	11 (13.3)	0.553
≥ 2 prior antibiotic treatments	64 (46.7)	39 (72.2)	25 (30.1)	< 0.001
Glucocorticoid therapy	77 (56.2)	40 (74.1)	37 (44.6)	0.001
Laboratory indicators, Median (IQR)				
WBC (×10^9^/L)	10.06 (6.07,15.88)	8.74 (5.05,13.79)	11.55 (7.12,18.02)	0.077
Neu (×10^9^/L)	8.86 (4.66,13.79)	7.38 (4.31,12.67)	9.67 (6.03,15.30)	0.112
Lym (×10^9^/L)	0.67 (0.38,1.23)	0.69 (0.41,1.19)	0.64 (0.32,1.24)	0.504
Hb (g/L)	77.00 (69.00,88.00)	77.00 (69.25,86.25)	78.00 (69.50,90.50)	0.247
CRP (mg/L)	75.91 (40.99,130.95)	53.83 (26.31,95.52)	88.43 (57.76,151.9)	< 0.001
PCT (ng/mL)	1.47 (0.43,3.83)	0.70 (0.42,2.09)	2.34 (0.48,6.25)	0.003
FIB (g/L)	3.53 (2.12,4.79)	2.82 (1.86,4.39)	3.96 (2.78,4.93)	0.005
BDG (pg/mL)	97.33 (37.50,210.85)	79.39 (37.5,140.52)	115.25 (41.69,335.07)	0.123
30-day mortality	26 (19.0)	10 (18.5)	16 (19.3)	1.000

*WBC* white blood cell count, *Neu* neutrophil count, *Lym* lymphocyte, *Hb* hemoglobin, *CRP* C-reactive protein, *PCT* procalcitonin, *FIB* fibrinogen, *BDG* (1,3)-*β*-D-glucan assay

### Comparison of clinical characteristics between patients with *C. auris* and non-auris *Candida* BSIs

Patients exhibited a significantly higher prevalence of hypertension (64.8% vs. 30.1%, *P* < 0.001) and cardiovascular disease (51.9% vs. 32.4%, *P* = 0.038) was observed in patients with *C. auris* BSIs compared with those with non-auris *Candida* infection. No statistically significant difference was observed between the two groups in other comorbidities, including diabetes and chronic pulmonary disease. Patients with *C. auris* BSIs more frequently required mechanical ventilation (85.2% vs. 49.4%, *P* < 0.001), were exposed to 2 or more antibiotics prior to infection (72.2% vs. 30.1%, *P* < 0.001), and received glucocorticoids therapy (74.1% vs. 44.6%, *P =* 0.001). In contrast, no statistically significant difference was observed in surgical history or prior use of echinocandins or triazole antifungal agents. Lower levels of C-reactive protein (CRP), procalcitonin, and fibrinogen were observed in the *C. auris* group compared with the non–auris *Candida* group (all *P* < 0.05). In contrast, no statistically significant difference was observed in white blood cell count, neutrophil count, hemoglobin, or (1,3)-*β*-D-glucan levels (Table 1). No significant difference in 30-day mortality was observed between the *C. auris* and non-auris *Candida* BSIs groups (18.5% vs. 19.3%, *P* = 1.000) (Table 1).

### Risk factors for *C. auris* BSIs

**Fig. 2:**
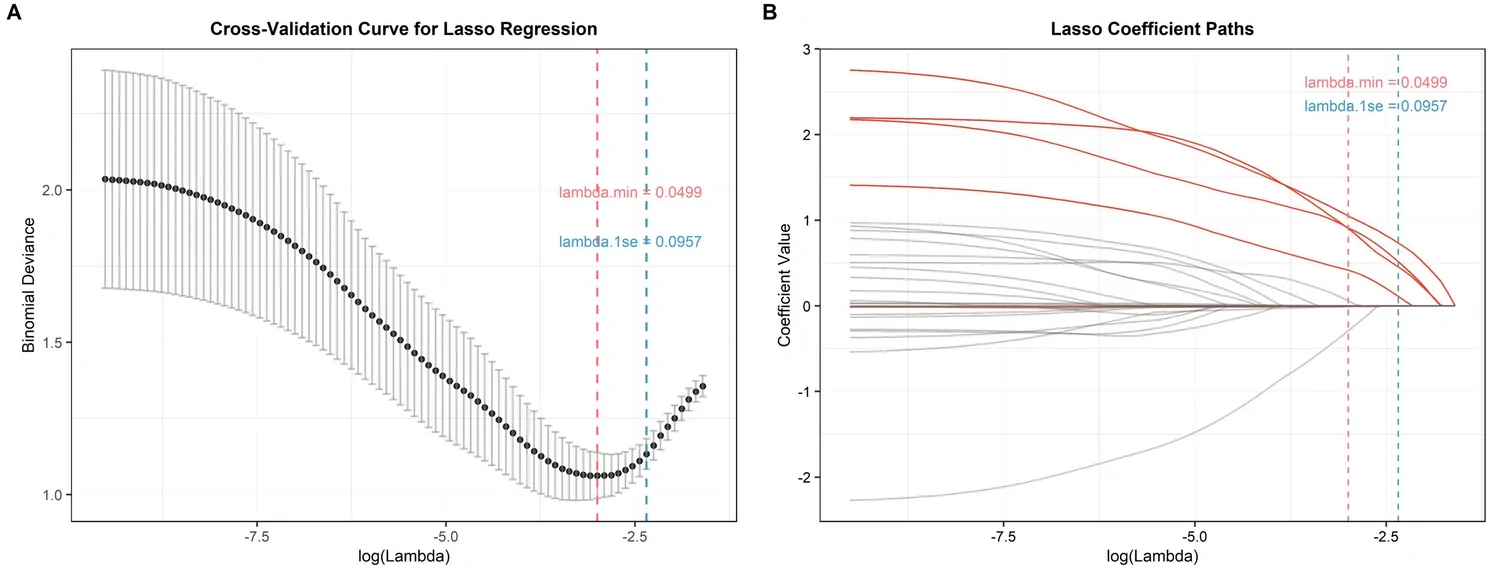
LASSO regression analysis for the selection of predictors of *Candida auris* BSIs. **A** Selection of the optimal parameter (lambda) in the LASSO model using tenfold cross-validation. Cross-validated deviance was plotted against log (lambda). **B** Coefficients profiles of the candidate predictors

Hypertension (OR = 6.283, 95% CI: 2.466–17.719), mechanical ventilation (OR = 4.329, 95% CI: 1.585–12.980), prior exposure to two or more antibiotics (OR = 5.991, 95% CI: 2.349–16.686), and glucocorticoid therapy (OR = 3.100, 95% CI: 1.196–8.509) were identified as independent risk factors for *C. auris* BSIs. In contrast, lower C-reactive protein (CRP) levels were independently associated with *C. auris* BSIs (OR = 0.987, 95% CI: 0.978–0.995) (Table 2; Fig. 2).

**Table 2:** Independent risk factors for *C. auris* BSIs identified by LASSO regression and confirmed by multivariable logistic regression

Variable	LASSO		Multivariate logistic	
	Coef	OR	OR (95% CI)	*P* value
Hypertension	0.517	1.677	6.283 (2.466–17.719)	< 0.001
Mechanical ventilation	0.457	1.579	4.329 (1.585–12.980)	0.004
≥ 2 prior antibiotic treatments	0.734	2.084	5.991 (2.349–16.686)	< 0.001
Glucocorticoid therapy	0.112	1.118	3.100 (1.196–8.509)	0.020
CRP	-0.002	0.998	0.987 (0.978–0.995)	< 0.001

### Distribution of *Candida* species and antifungal susceptibility

Among the 137 candidemia cases, 54 (39.4%) were attributed to *C. auris*, while the remaining 83 (60.6%) were caused by non-auris *Candida*. *C. auris* was the most frequently isolated species (39.4%), followed by *Candida albicans* (*C. albicans*, 27.7%), *Candida tropicalis* (*C. tropicalis*, 12.4%), and *Nakaseomyces glabratus* (*N. glabratus*, 10.9%). As per CDC tentative breakpoints, *C. auris* exhibited complete resistance to fluconazole (100%) and showed an evident resistance to AMB (21%). Fluconazole resistance was also observed in *C. tropicalis* (36.8%) and *C. parapsilosis* (*C. parapsilosis*, 20%), suggesting a species-specific variability in susceptibility patterns. No resistance to echinocandins was observed among most *Candida* species except for *C. tropicalis* (Table 3). The antifungal susceptibility profile of *C. auris* to various antifungal agents is summarized in Table 4.

**Table 3:** Distribution of *Candida* species and antifungal resistance

Species	Number	(%)	CAS (*R*%)	MIF (*R*%)	ANI (*R*%)	AMB (*R*%)	FLU (*R*%)	VOR (*R*%)
*C. auris*	54	39.4	0	0	0	21	100	/
*C. albicans*	38	27.7	0	0	0	/	0	5
*C. tropicalis*	17	12.4	5.3	5.3	5.3	/	36.8	36.8
*N. glabratus*	15	10.9	0	0	0	/	0	/
*C. parapsilosis*	11	8.0	0	0	0	/	20	13.7
*P. kudriavzevii*	2	1.5	0	0	0	0	100	0

*CAS* caspofungin, *MIF* micafungin, *ANI* anidulafungin, *AMB* amphotericin B, *FLU* fluconazole, *VOR* voriconazole, *C. albicans Candida albicans*, *C. tropicalis Candida tropicalis*, *N. glabratus Nakaseomyces glabratus*, *C. parapsilosis Candida parapsilosis*, *P. kudriavzevii Pichia kudriavzevii*

**Table 4:** MIC of 54 *C. auris* isolates against 5 antifungal drugs

	CAS	MIF	ANI	AMB	FLU
S%	100.0	100.0	100.0	79	0.0
R%	0	0	0	21	100
MIC_50_	0.125	0.125	0.125	1	128
MIC_90_	0.5	0.5	0.5	2	256
Range	0.06–1	0.03–2	0.03–2	0.5–4	32–256

*MIC*_*50*_ MIC at which 50% of test isolates were inhibited, *MIC*_*90*_ MIC at which 90% of test isolates were inhibited, *CAS* caspofungin, *MIF* micafungin, *ANI* anidulafungin, *AMB* amphotericin B, *FLU* fluconazole, *VOR* voriconazole

## Discussion

*C. auris* was identified as the most frequently isolated species among candidemia cases in Southern China, surpassing *C. albicans*, suggesting a shift in the local epidemiology landscape of invasive candidiasis. Independent risk factors identified in this study included hypertension, mechanical ventilation, prior exposure to two or more antibiotics, and glucocorticoid therapy, highlighting the association of *C. auris* BSIs with both underlying comorbidities and intensive clinical interventions in this region.

Patients with *C. auris* BSIs exhibited clinical characteristics broadly comparable to those with non-auris *Candida* infections in terms of demographic features, while significant differences were observed in comorbidity burden and treatment-related exposures. *C. auris* infections were more frequently observed in older and male patients, consistent with previous reports [18–20]; however, the biological basis for this sex-related difference remains unclear [21]. Higher rates of mechanical ventilation and glucocorticoid therapy were observed in the *C. auris* group, suggesting an association with more severe clinical conditions. Previously reported risk factors for *C. auris* BSIs have varied across studies. Ruiz-Gaitán et al. identified prior antibiotic exposure, invasive devices, surgical history, and mechanical ventilation as crucial risk factors [11], whereas another study emphasized prolonged ICU stay and excessive antifungal use [10]. As a tertiary referral center for critically ill patients, our hospital frequently treats complex cases, leading to the common empirical use of echinocandins upon admission for patients at risk of multidrug-resistant *Candida* infections. Consequently, the high prevalence of prior antifungal exposure in our cohort is largely driven by this early empirical therapy. Such variability likely reflects differences in study design, patient populations, sample sizes, and data completeness. Potential mechanisms, including endothelial dysfunction and vascular remodeling, contribute to increased susceptibility, although further studies are required to confirm this hypothesis [22].

The inverse association between CRP levels and *C. auris* infection should not be interpreted as evidence of a protective effect. Instead, it may reflect a distinct host inflammatory response to *C. auris* compared with that elicited by other *Candida* species. Bruno et al. [23] demonstrated that *C. auris* induces a specific transcriptional response in human mononuclear cells, eliciting a stronger pro-inflammatory cytokine response than *C. albicans* while exhibiting a lower capacity to induce macrophage lysis. Mechanistically, this innate immune response is mediated by C-type lectin receptors and structurally unique mannoproteins, suggesting pathogen-specific host recognition mechanisms. Furthermore, *C. auris* induces a distinct cytokine profile that lies between those induced by fluconazole-susceptible and fluconazole-resistant strains, displaying a unique immunophenotype [24]. As an acute-phase protein whose production is driven by IL-6, CRP may be affected by pathogen-specific modulation of the host inflammatory response. Thus, the lower CRP levels observed in patients with *C. auris* infections may not necessarily indicate attenuated virulence or a more favorable clinical outcome. Notably, the levels of other inflammatory markers, including procalcitonin and fibrinogen, were also lower in this study, further suggesting that *C. auris* infection may elicit a distinct systemic host response. Although Bruno et al. [23] showed that *C. auris* is less virulent than *C. albicans* in a mouse model, mortality associated with C. auris infection in clinical settings remains high. Therefore, this distinct inflammatory profile should not be interpreted as a protective signal.

Despite more complex clinical profiles, no significant difference in 30-day mortality was observed between patients with *C. auris*. However, this finding should be interpreted with caution, as the absence of a significant difference may reflect survivor bias rather than equivalent mortality risk. This observation differed from previous international reports describing higher mortality rates [7, 25] and may be influenced by differences in clinical management or patient characteristics; however, causal inferences could not be established from the present results.

All *C. auris* isolates were resistant to fluconazole, confirming the high level of antifungal resistance associated with this pathogen, consistent with global reports [26, 27]. AMB susceptibility testing presents inherent technical challenges, including poor water solubility, instability in standard fungal culture media, which may particularly affect slow-growing isolates, and marked sensitivity of MIC measurements to variations in inoculum size, medium composition, and incubation temperature [28, 29]. Moreover, many commercial testing platforms tend to overestimate AMB resistance [30, 31]. In our study, 21% of isolates had MICs above the tentative breakpoint. This proportion was lower than the approximately 40% reported in some studies [10, 32], but nevertheless indicated reduced susceptibility in a substantial proportion of isolates. Given the methodological limitations described above, however, these results should be interpreted with caution. No resistance to echinocandin was detected, supporting their continued clinical use as first-line therapy. However, interpretation of susceptibility results remains challenging due to the absence of standardized clinical breakpoints for *C. auris*. Although fluconazole and voriconazole share overlapping resistance mechanisms, cross-resistance is not absolute because resistance profiles depend on the specific determinants expressed and may vary substantially even among strains of the same species [33, 34]. This observation highlights the complexity of azole resistance and supports isolate-specific susceptibility testing for individual antifungal agents.

The high rate of azole resistance in *C. tropicalis* and the emerging resistance in *C. parapsilosis* are additional causes for concern. The fluconazole resistance rate in *C. tropicalis* far exceeds the reported global range of 0%-18% [35], highlighting a potentially important regional challenge. Although primary resistance is uncommon in this species, resistance may emerge following drug exposure [36]. Extensive clinical use of azoles, particularly for prophylaxis in hematopoietic stem cell transplant recipients [37], may contribute to this trend. Although fluconazole resistance in *C. parapsilosis* remained low, its recent increase, together with the risk of heteroresistance and nosocomial outbreaks [38], represents an emerging threat. Collectively, these findings reinforce the need for routine antifungal susceptibility testing to guide treatment, particularly in settings with high resistance rates like those in China [39].

Several limitations should be acknowledged. First, we did not perform whole-genome sequencing (WGS) or targeted analyses of resistance-associated mutations because of the retrospective study design and resource constraints. Consequently, we were unable to determine the specific clade (I-VI) or identify genetic mutations associated with antifungal resistance. Future multicenter studies incorporating genomic characterization are urgently needed to elucidate the molecular epidemiology of *C. auris* in Southern China. Second, the single-center retrospective design may introduce selection bias, potentially limiting the generalizability of the findings to broader populations or other geographic regions. Third, the relatively small sample size may reduce statistical precision, as reflected by the wide confidence intervals observed for some variables, including hypertension. Further multicenter and prospective studies are required to validate these findings.

## Conclusion

*C. auris* was identified as the predominant cause of candidemia in Southern China, indicating a shift in the local epidemiological landscape. Hypertension, mechanical ventilation, prior exposure to two or more antibiotics, and glucocorticoid therapy were identified as independent risk factors for *C. auris* bloodstream infections. Lower CRP levels were independently associated with *C. auris* BSIs rather than BSIs caused by other *Candida* species; however, this inverse association should not be interpreted as evidence of a causal relationship or protective effect. These findings contribute to the current understanding of the clinical risk profile of *C. auris* and support the development of targeted prevention and management strategies. Further prospective multicenter studies with genomic characterization are required to validate these results.

## Data Availability

Data will be available upon reasonable request from the corresponding author Pingjuan Liu, Email: liupj8@mail.sysu.edu.cn).

## Abbreviations

*C. auris*: *Candidozyma auris*
*C. albicans*: *Candida albicans*
*C. tropicalis*: *Candida tropicalis*
*N. glabratus*: *Nakaseomyces glabratus*
*C. parapsilosis*: *Candida parapsilosis*
*P. kudriavzevii*: *Pichia kudriavzevii*
MIC: Minimum Inhibitory Concentrations
VOC: Voriconazole
FLU: fluconazole
CAS: Caspofungin
MIF: Micafungin
ANI: Anidulafungin
AMB: Amphotericin B
WBC: White blood cell count
Neu: Neutrophil count
Lym: Lymphocyte
Hb: Hemoglobin
CRP: C-reactive protein
PCT: Procalcitonin
FIB: Fibrinogen
BDG: (1,3)-β-D-glucan assay
BSIs: Bloodstream infections
